# Candida tropicalis-Associated Osteomyelitis in an Intravenous Drug User: A Case Report

**DOI:** 10.7759/cureus.87438

**Published:** 2025-07-07

**Authors:** Chukwunonso B Ubanatu, John K Appiah, Connor Barry

**Affiliations:** 1 Internal Medicine, Geisinger Health System, Wilkes Barre, USA

**Keywords:** antifungal susceptibility, candida tropicalis, discitis-osteomyelitis, drug addiction, intravenous drug user

## Abstract

*Candida* species represent the most frequent fungal cause of osteomyelitis in patients with a history of drug use, with *Candida albicans* historically being the predominant pathogen. However, non-albicans *Candida* species, particularly *Candida tropicalis*, have emerged as increasingly important pathogens associated with more complex clinical presentations and treatment challenges. *Candida tropicalis* (*C. tropicalis)* demonstrates enhanced virulence factors, including superior adherence capabilities and biofilm formation, contributing to its pathogenicity in immunocompromised hosts and those with indwelling foreign materials.

We present a case of a 36-year-old man who presented with severe back pain, unrelieved with pain medication (ibuprofen 400mg daily). He endorsed heroin use a month prior, and magnetic resonance imaging (MRI) of the lumbar spine revealed possible osteomyelitis/discitis of the lumbar spine, which required a biopsy of the lumbar spine. Biopsy results later revealed osteomyelitis, with sensitivities positive for* C. tropicalis*. The patient was discharged home on fluconazole 800 mg daily for six months with follow-up arranged as an outpatient with infectious disease and addiction medicine. This case highlights the diagnostic challenges, treatment considerations, and addiction medicine implications of this uncommon but serious complication of candida infection. This case also underscores the importance of maintaining a high index of suspicion for fungal pathogens in intravenous drug use (IVDU)-related bone and joint infections, particularly when initial bacterial cultures are negative.

## Introduction

*Candida tropicalis* (*C. tropicalis*) is a species of yeast in the genus *Candida*, and the global impact of candidiasis has increased globally [1]. *C. tropicalis* exhibits high virulence due to its ability to form biofilms, secrete lytic enzymes, adhere to epithelial and endothelial cells, and undergo transition of bud to hyphae [2]. Conditions that contribute to *C. tropicalis* colonization are increased antifungal and antibacterial regimens, immunocompromised, and long-term use of catheters [1]. 

Osteomyelitis in intravenous drug users (IVDU) presents unique diagnostic and therapeutic challenges, with bacterial pathogens such as *Staphylococcus aureus* and *Pseudomonas aeruginosa* representing the majority of cases. However, fungal osteomyelitis, while uncommon, is increasingly recognized as a serious complication of injection drug use [3]. Recent systematic reviews demonstrate that fungal osteomyelitis affects both immunocompetent and immunocompromised patients, with *Candida *species accounting for 20.7% of all fungal osteomyelitis cases, representing a disproportionately higher prevalence among IVDU populations compared to the general population [3]. Candida osteomyelitis most frequently presents subacutely with local symptoms but minimally elevated inflammatory biomarkers, following a more indolent course, with subtle symptoms that may delay diagnosis for weeks to months compared to bacterial infections [4]. This diagnostic delay is particularly problematic in IVDU populations, where healthcare engagement may be intermittent, and presenting symptoms such as chronic pain or low-grade fever may be attributed to withdrawal, intoxication, or other substance-related complications [3,4].

Vertebral osteomyelitis is most common in adults with candidal bone infections, while vertebral osteomyelitis remains rare among invasive candidiasis complications, with most cases caused by* C. albicans* followed by less common organisms, including *C. tropicalis *[4]. Despite *Candida* being part of normal flora, it represents the fourth leading cause of hematogenous nosocomial infections, making recognition vital in high-risk populations [5]. Definitive diagnosis is made by bone biopsy, performed after imaging confirms infection in the vertebrae [5]. Treatment typically involves intravenous antifungal induction followed by prolonged therapy with oral fluconazole. However, the prolonged course of antifungal therapy, potential for significant drug interactions, and need for consistent medical follow-up pose particular challenges in populations that may have limited healthcare access and competing priorities related to addiction recovery [6].

## Case presentation

A 36-year-old man with a history of intravenous heroin use, major depressive disorder, and anxiety presented to the emergency department with acute-onset lower back pain and fever. He reported a five-day history of severe lower back pain associated with fever and chills. He described the pain as constant, aching, and localized to the lumbar region without radiation. The pain was exacerbated by movement and was not relieved by over-the-counter analgesics. He denied any neurological symptoms, including weakness, numbness, or bowel/bladder dysfunction. The patient reported last using heroin approximately one month prior to presentation. His medical history was significant for hepatitis C infection, but he denied any history of endocarditis, previous spine infections, or recent hospitalizations. He was not taking any regular medications and reported no known drug allergies.

On physical examination, the patient appeared acutely ill and was in moderate distress due to pain. Vital signs were notable for fever (temperature 38.9°C), tachycardia (heart rate 110 bpm), and normal blood pressure (128/76 mmHg). Examination of the lumbar spine revealed tenderness to palpation over the L3-L4 region with limited range of motion due to pain. No erythema or swelling was observed. Neurological examination was intact with normal strength, sensation, and reflexes in the lower extremities. Cardiac examination revealed tachycardia with regular rhythm and no murmurs. The remainder of the physical examination was unremarkable, with no evidence of injection site infections or other stigmata of endocarditis.

Initial laboratory studies revealed leukocytosis with an elevated white blood cell count and a normal hemoglobin. Inflammatory markers (C-reactive protein (CRP) and erythrocyte sedimentation rate (ESR)) were significantly elevated. Liver function tests showed mild transaminase elevation (Table 1).

**Table 1:** laboratory values on admission. WBC, white blood cells); Hb; hemoglobin; CRP, C-reactive protein; ESR, erythrocyte sedimentation rate; ALT, alanine aminotransferase; AST, aspartate aminotransferase; ALP, alkaline phosphatase

Parameter	Value	Reference range
WBC	14.93	4.0-11.0 × 10³/μL
Hb	15.1	14-16.8 g/dL
CRP	77	<3.0 mg/L
ESR	71	0-15 mm/hr (for males)
ALT	67	7-56 U/L
AST	55	10-40 U/L
ALP	221	44-147 U/L

Hepatitis C antibody was positive, and quantitative HCV RNA was elevated at 863,000 IU/mL, consistent with chronic hepatitis C infection. Two sets of blood cultures were obtained on admission and remained negative for bacterial and fungal growth throughout hospitalization.

**Figure 1:**
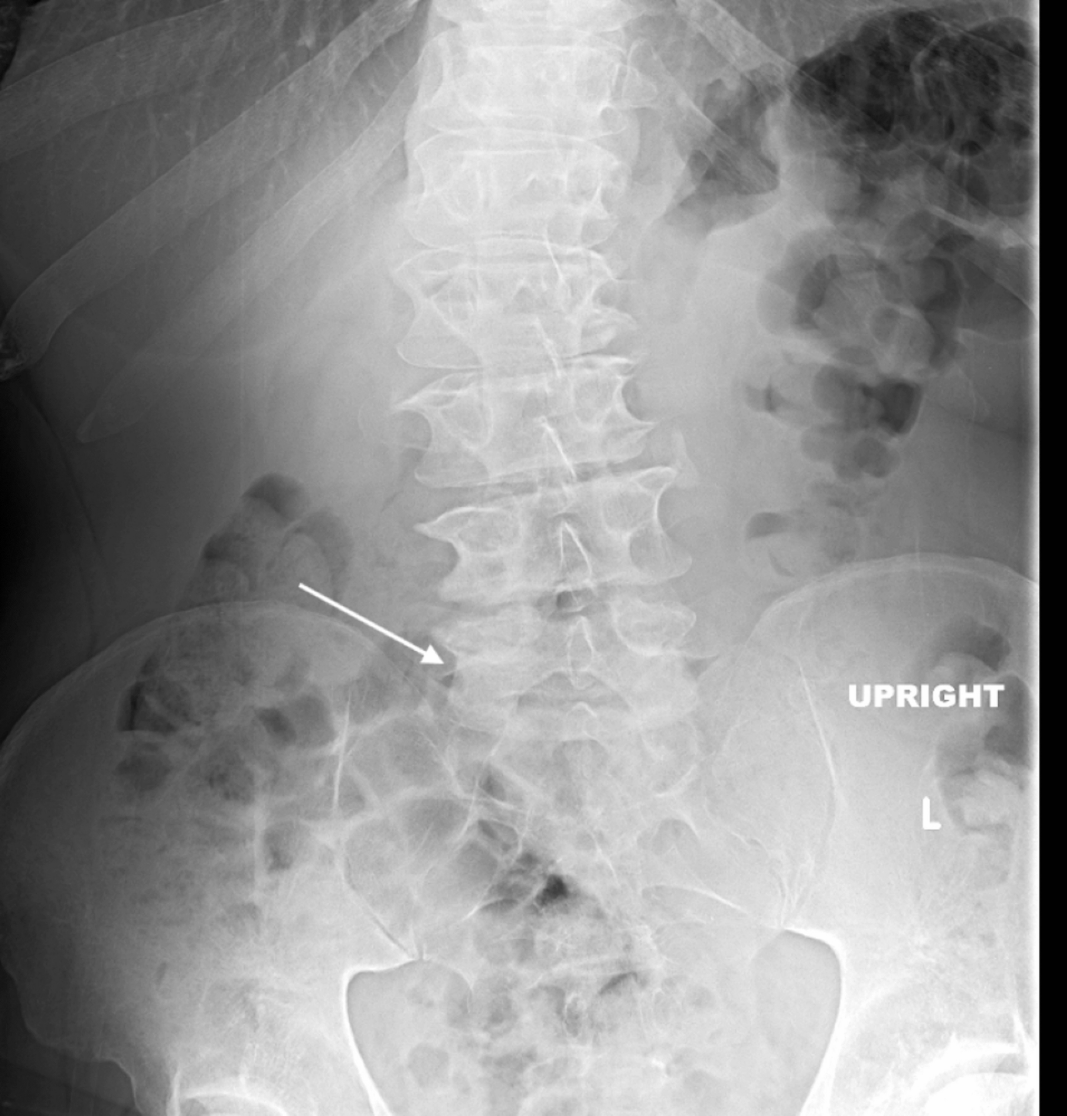
X-ray of the lumbar spine showing only degenerative changes (white arrow). The absence of evidence of the ongoing spinal infection is notable.

Plain radiographs of the lumbar spine demonstrated degenerative changes without evidence of paravertebral soft tissue swelling or effusion (Figure 1).

**Figure 2:**
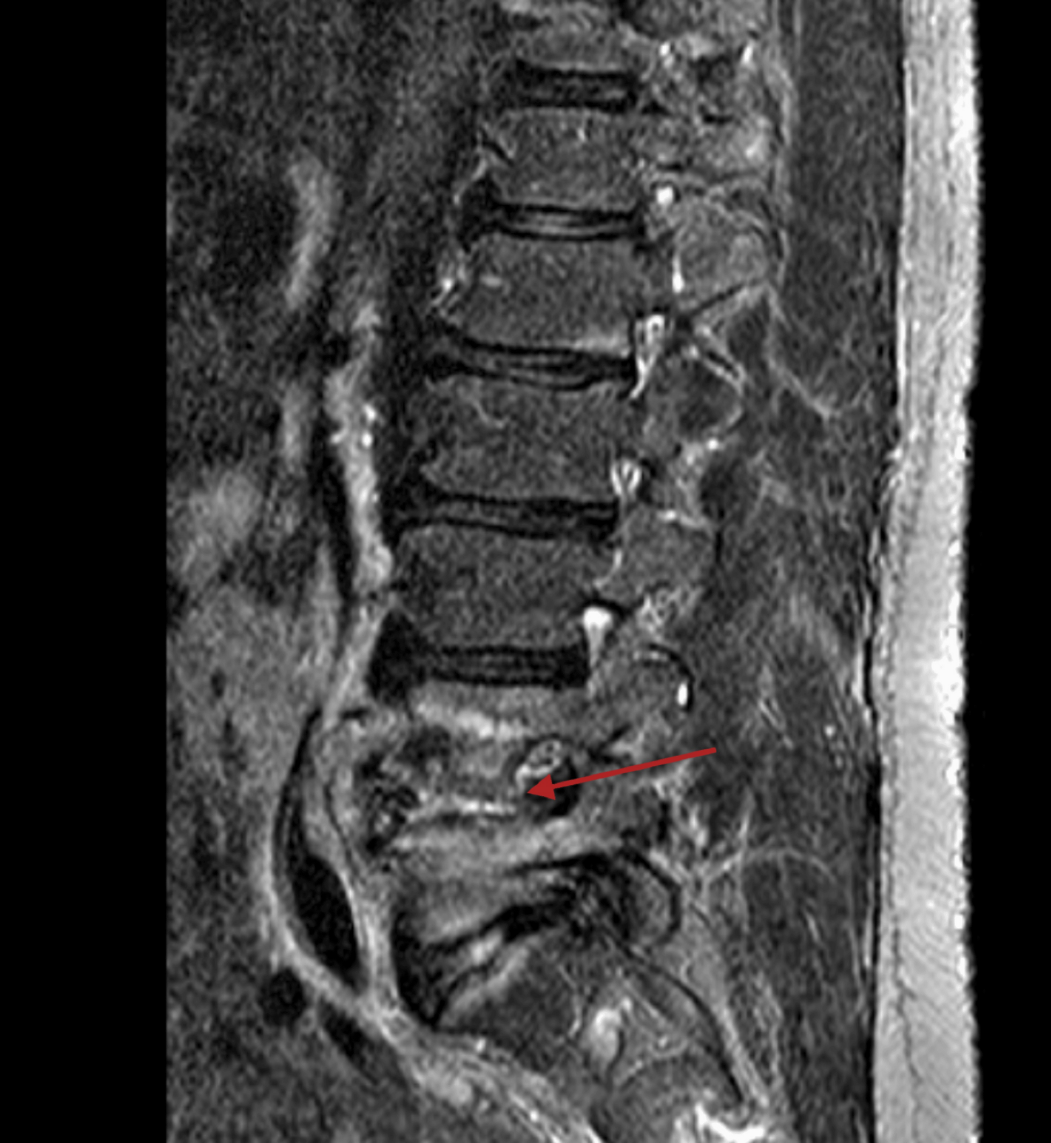
MRI lumbar spine with and without contrast (SAG STIR series) demonstrating osteomyelitis at L4-L5, with potential marrow edema (red arrow). Disc protrusion is also seen at L5-S1. There is central stenosis at L3-L4 (mild to moderate), L4-L5 (moderate to marked), and L5-S1 (moderate).

Given the high clinical suspicion for vertebral osteomyelitis, magnetic resonance imaging (MRI) of the lumbar spine was obtained, which revealed findings consistent with discitis and osteomyelitis at the L4-L5 level. The MRI showed worsening marrow edema involving both L4 and L5 vertebral bodies with associated disc space narrowing and enhancement, confirming the diagnosis of spondylodiscitis (Figure 2).

Human immunodeficiency virus(HIV) serology was negative. Given the patient's history of intravenous drug use and concern for possible hematogenous seeding, transthoracic echocardiography was performed to evaluate for infective endocarditis. The echocardiogram showed normal cardiac structure and function without evidence of valvular vegetations, effectively ruling out endocarditis.

Given the clinical presentation suggestive of vertebral osteomyelitis and negative blood cultures, a CT-guided lumbar spine biopsy was performed at the L4-L5 level. Histopathological examination of the bone tissue revealed chronic inflammation with the presence of yeast forms consistent with fungal infection. Fungal culture of the biopsy specimen grew *C. tropicalis* after 48 hours of incubation. The isolate was sent for antifungal susceptibility testing to guide targeted therapy (Table 2). 

**Table 2:** Susceptibilities from the culture growth of Candida tropicalis obtained from lumbar spine biopsy. Microbroth dilutions were used for testing.

Drug	Susceptibility
Amphotericin B	No interpretation
Fluconazole	Susceptible
Isavuconazole	No interpretation
Micafungin	Susceptible
Voriconazole	Susceptible

Based on the susceptibility results, the patient was initially started on intravenous (IV) micafungin 100 mg daily for the treatment of Candida tropicalis vertebral osteomyelitis. After six days of inpatient treatment with clinical improvement (stable vital signs, minimal back pain), the decision was made to transition to oral therapy given the patient's history of intravenous drug use and the need for prolonged treatment. It is to be noted that neurosurgery was consulted, but they declined surgical intervention and recommended medical management only, as the patient had no red flag symptoms (fever, weight loss, structural deformity, pain radiating down the knee, decreased anal sphincter tone, urinary or fecal incontinence). The patient was switched to oral fluconazole 800 mg daily on discharge, with a planned duration of six months of total antifungal therapy. Pain management was provided with oral analgesics and anti-inflammatory medications. The patient was counseled on substance abuse treatment options and provided with resources for addiction recovery services. The treatment plan includes weekly monitoring of inflammatory markers (ESR and CRP) for six weeks post-discharge to assess treatment response, with repeat MRI of the lumbar spine scheduled at six weeks post-hospital discharge to evaluate radiographic improvement.

## Discussion

*C. tropicalis* is recognized as one of the five most common *Candida* species causing invasive disease, accounting for a significant proportion of non-albicans candidemia. The epidemic of illicit intravenous drug use (IVDU) in the United States has been accompanied by a surge in infectious complications, with Candida infections being a notable sequela [7]. Patients with a history of IVDU are more likely to have non-albicans Candida infections, be co-infected with hepatitis C, and develop end-organ involvement, including endocarditis and osteomyelitis [7].

Recent surveillance data demonstrate the reemergence of IVDU as a significant risk factor for candidemia. Surveillance for candidemia across nine US states during 2017 revealed that 10.7% of cases involved recent injection drug use, representing an unexpectedly high proportion [8]. Injection drug use and previous spine surgery were identified as the two most common risk factors for vertebral osteomyelitis due to *Candida* species [8,9]. Users of illicit intravenous drugs develop a distinctive syndrome consisting of a febrile illness with disseminated cutaneous, follicular, nodular, ocular, and osteoarticular lesions [8,9]. The pathogenesis likely involves hematogenous seeding from contaminated injection equipment or drug preparations, with subsequent dissemination to bone and joint structures [10].

Candida osteomyelitis should be considered when a patient presents with risk factors and pain without previous trauma, as candidal infections constitute the fourth leading cause of hematogenous nosocomial infections [10]. The diagnosis of fungal osteomyelitis remains challenging due to the nonspecific clinical presentation and the frequent occurrence of negative blood cultures, as demonstrated in our case. The superior sensitivity of MRI over plain radiography for detecting early osteomyelitis is well-established, and tissue biopsy remains the gold standard for diagnosis when blood cultures are negative [11]. The recommended treatment approach includes surgery and fluconazole as first-line therapy for susceptible isolates. Treatment typically consists of intravenous antifungal induction followed by prolonged therapy with oral fluconazole [11].

The decision to transition from intravenous micafungin to oral fluconazole in our case was influenced by several factors: demonstrated susceptibility to fluconazole, the patient's history of IVDU (which poses risks for long-term intravenous access), and the need for prolonged antifungal therapy.

*C. tropicalis *exhibits variable antifungal susceptibility patterns, making susceptibility testing crucial for optimal treatment selection [12]. The isolate in our case demonstrated susceptibility to fluconazole, micafungin, and voriconazole, providing multiple therapeutic options. The lack of interpretive criteria for amphotericin B reflects evolving laboratory standards and the shift toward newer antifungal agents with better tolerability profiles [13].

## Conclusions

*C. tropicalis* is a rare but important cause of osteomyelitis, particularly in those who inject drugs. This case highlights several important clinical considerations for healthcare providers managing patients with IVDU histories. The increasing prevalence of injection drug use-associated candidemia necessitates heightened clinical suspicion for invasive candidiasis in this population, especially when there is a lack of response to broad-spectrum antibiotics. Early diagnosis through appropriate imaging and microbiological evaluation, followed by timely antifungal therapy and surgical management (when necessary), are key to improving outcomes. Increased awareness of such atypical presentations can aid in reducing diagnostic delays and guide treatment strategies. Patients need to be adequately followed up by the proper specialists, for example, addiction medicine specialists and counselors, to reduce relapse and improve the overall outcome.
